# Acute appendicitis due to appendiceal endometriosis: Two case report and literature review

**DOI:** 10.1016/j.ijscr.2024.110743

**Published:** 2024-12-15

**Authors:** Abdala Bolcatto, Melisa Erina, Facundo Ignacio Mandojana, Nicolás Bruera, Alejandro Marcelo Doniquian, German Rodrigo Viscido

**Affiliations:** Clinica Universitaria Reina Fabiola, Córdoba, Argentina

**Keywords:** Appendiceal endometriosis, Acute appendicitis, Laparoscopic appendectomy

## Abstract

**Introduction:**

Appendiceal endometriosis (AE) is a rare condition, with a prevalence ranging from 0.05 % to 1.7 % in patients with endometriosis. It represents <1 % of cases of acute appendicitis (AA).

**Cases presentation:**

We present two cases of AA where the histological cause was endometriosis. Both cases involved patients around 40 years old who presented with abdominal pain in the right iliac fossa. AA was diagnosed through abdominal computed tomography (CT), which in the first case showed acute appendicitis, successfully treated with laparoscopic appendectomy. In the second case, the CT showed signs of an appendiceal phlegmon, initially treated non-operatively with poor response, leading to exploratory laparoscopy and abscess drainage 48 h later. Subsequently, a scheduled laparoscopic appendectomy was performed after 6 months. Histopathological diagnosis in both cases was AA due to AE with endometrial glands showing recent bleeding, causing hyperplasia of the appendiceal muscular layer.

**Discussion:**

Endometriosis, characterized by the presence of endometrial tissue outside the uterine cavity, can rarely affect the appendix, termed AE. AE, though uncommon, poses diagnostic challenges due to nonspecific imaging findings and variable presentations, ranging from asymptomatic cases to AA. Histological evaluation post-appendectomy is definitive for diagnosis. AE is associated with right-sided pelvic involvement and often requires surgical management, with appendectomy typically resolving acute symptoms. However, recurrence of cyclical pain due to pelvic endometriosis may persist, underscoring the importance of comprehensive evaluation during laparoscopic procedures.

**Conclusion:**

AA caused by AE is an uncommon condition, with very difficult preoperative diagnosis based solely on personal history, clinical presentation, and even imaging studies. It should be considered in differential diagnoses for women of reproductive age with associated pelvic endometriosis, although the recommended treatment in all cases is surgical.

## Highlights

• Appendices endometriosis (AE) is a rare condition and represents less than 1% of cases of acute apendicitis (AA).
• It presents as abdominal pain, mimicking an AA.
• AE should be considered as a differential diagnosis in women of reproductive age, more so if the have a history of endometriosis.
• Preoperative diagnosis is challenging and the definitive diagnosis is histological.
• The appendenctomy, laparoscopically or not, is the ultimate treatment.

## 1 Introduction

Endometriosis is the presence of endometrial tissue outside the uterine cavity and affects 6–10 % of women of reproductive age. It is most commonly located in the ovary and fallopian tube [1,2].

Less frequently, it can be found in the gastrointestinal tract, urinary system, soft tissues, and thorax, among others [[2], [3], [4]]. Intestinal localization is variable, occurring in 3–37 % of cases. The sigmoid colon and rectum are the most frequently affected sites, with less frequent involvement of the small intestine, peritoneum, diaphragm, cecum, and appendix [2,4,5].

Appendiceal endometriosis (AE) is an unusual location, with a prevalence of 0.05–1.7 % in women with known endometriosis, although this percentage increases in patients with deep endometriosis or those investigated for chronic pelvic pain [[6], [7], [8], [9]]. AE can be asymptomatic or present as cyclical abdominal pain in the lower abdomen. It may also manifest as acute appendicitis (AA) or chronic appendicitis, gastrointestinal bleeding, intussusception, intestinal perforation, or as a tumor [2,[9], [10], [11]].

AA due to AE is extremely rare, constituting 0.5–2.5 % of all causes of acute appendiceal pathology [7,9,[11], [12], [13]].

Despite the high sensitivity and specificity of imaging studies for AA diagnosis, it is anecdotal that they can provide a preoperative etiological diagnosis for AA caused by endometriosis. Currently, laparoscopic exploration is the standard for direct visualization and resection of the specimen for definitive histological diagnosis [2,[12], [13], [14], [15]].

We present two cases of acute appendicitis caused by endometriosis.

## 2 1st case presentation

A 41-year-old female with a history of cesarean section presented to the emergency department with continuous abdominal pain for 12 h, localized in the right iliac fossa (RIF). She had no fever or genitourinary symptoms. She was on day 20 of her menstrual cycle, with regular monthly cycles. No dysmenorrhea or cyclical pelvic pain.

Physical examination revealed abdominal pain in RIF, with localized tenderness in the RIF but no rebound tenderness was found. McBurney's point was positive and the obturator sign was negative.

Laboratory results showed white blood cells at 8800/mm^3^ (normal range (NR) 3800–10,000/mm^3^), C-reactive protein (CRP) at 55 mg/L (NR <5 mg/L), erythrocyte sedimentation rate at 29 mm/h (NR 3–20 mm/h), negative chorionic gonadotropin, and other values within normality.

Abdominal and pelvic ultrasound showed only free fluid in the Pouch of Douglas (POD). Abdominal CT with intravenous contrast (Image 1A, Image 1B) revealed an enlarged cecal appendix measuring 8 mm in thickness with inflammatory changes, and an enlarged right ovary measuring 4 cm.

**Image 1A:**
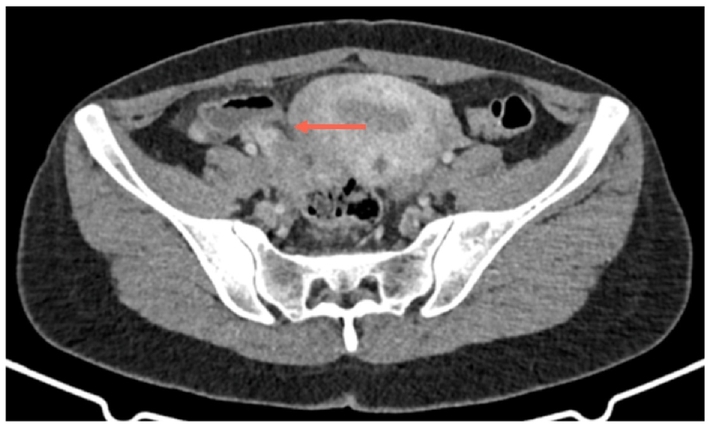
Enlarged appendix with right adnexa enlarged as well.

**Image 1B:**
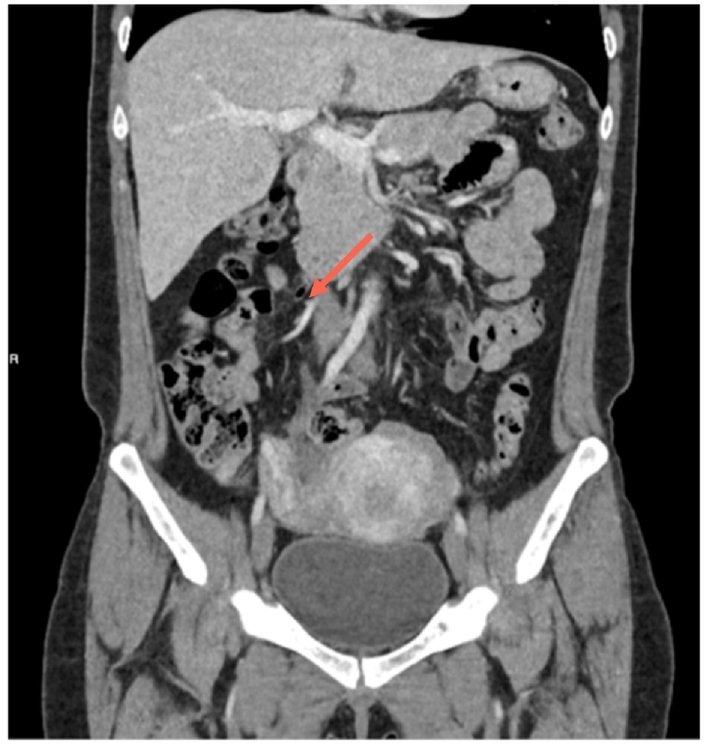
Enlarged appendix with right adnexa enlarged as well.

Laparoscopic exploration was decided, which revealed phlegmonous appendicitis without peritonitis, showing the appendix swollen, erythematous and thickened, with purulent material in the lumen and fibrinous exudates on the serial surface; and concurrent inflammatory congestion of the right fallopian tube. A laparoscopic appendectomy was performed successfully, and gynecology was consulted for a right salpingectomy due to the inflammatory process. No suggestive foci of gynecological or abdominal endometriosis were observed.

The patient had an uneventful postoperative course and was discharged 24 h later.

**Image 2:**
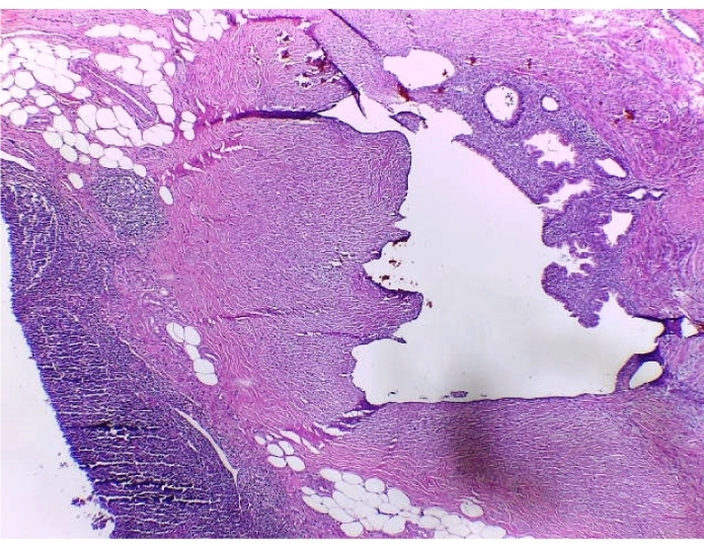
Endometrial foci on the external muscular layer. HE 10×.

**Image 3:**
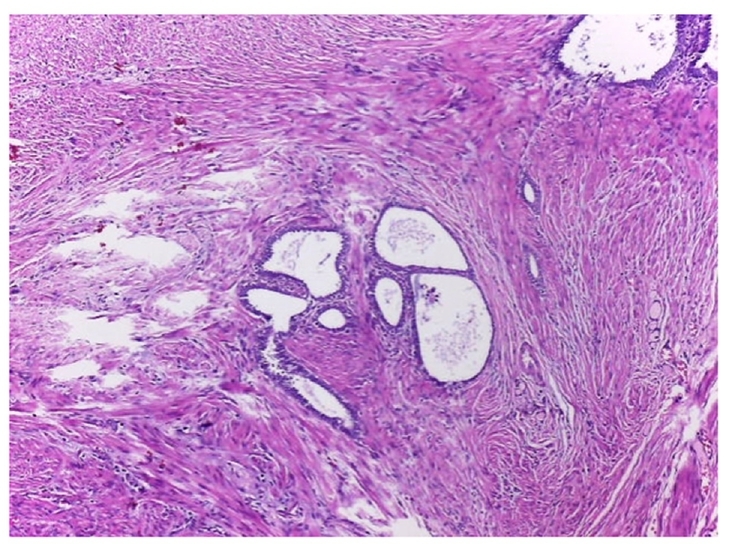
Another endometrial foci on the external muscular layer. HE 10×.

**Image 4:**
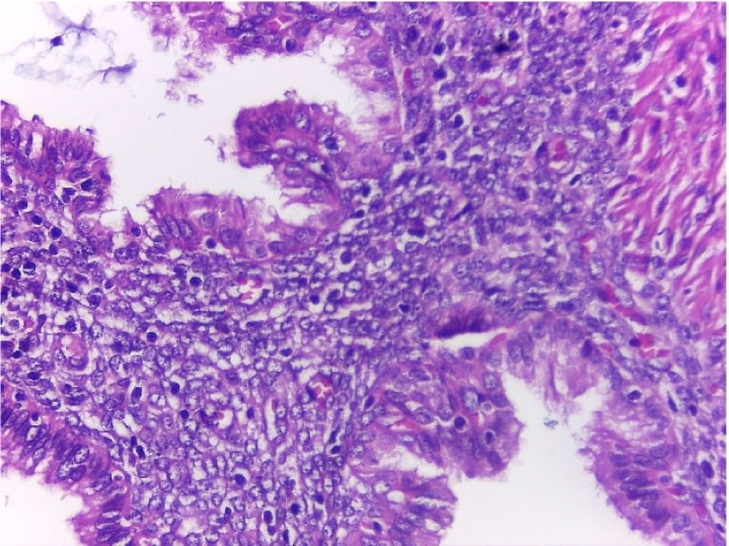
Endometrial foci. Cylindrical epithelium and endometrial stroma. HE 40×.

Histopathology of the appendix describes the presence of ectopic endometrial stroma within the muscularis layer of the appendix, a hallmark of endometriosis; it revealedsigns of recent bleeding that suggests active involvement, consistent with one of the characteristic features of endometriotic tissue, which includes hemorrhage from ectopic endometrial glands and stroma; it also described endometrial stroma within the muscular layer (Image 2, Image 3, Image 4). The fallopian tube histology showed endometrial glands and stroma, negative for malignancy.

At 6 month follow-up, the patient remains asymptomatic with follow-up in gynecology.

## 3 2nd case presentation

A 44-year-old female with a history of endometriosis on hormonal treatment, previous surgeries including two cesarean sections and a laparoscopic exploration with resection of pelvic endometriotic foci two years prior, presented with abdominal pain for 72 h, initially in the epigastrium and later localized in RIF, associated with oral intolerance. No fever or urinary symptoms. No dysmenorrhea or chronic cyclical abdominal pain.

Physical examination showed abdominal pain in RIF, with localized tenderness and positive McBurney's sign. Rebound tenderness was positive in the RIF showing a positive Blumberg sign; obturator sign was negative.

Laboratory tests indicated leukocytosis with 17,200/mm^3^ white blood cells (NR 3800–10,000/mm^3^), CRP at 85 mg/L (NR <5 mg/dL). A presumptive diagnosis of acute appendicitis was made, and an abdominal and pelvic CT revealed an enlarged cecal appendix measuring 17 mm in diameter with marked inflammatory process around the cecum and terminal ileum, suggestive of an appendiceal phlegmon.

With a diagnosis of advanced appendicitis, non-operative treatment with intravenous antibiotics was initially indicated. The evolution was irregular, with fever, increased abdominal pain, tachycardia, and rising acute phase reactants, leading to exploratory laparoscopy which revealed inflammatory process in RIF adhered to the anterior and lateral parietal peritoneum involving the cecum, terminal ileum, and greater omentum. A 75 cc abscess of purulent fluid was drained. No further surgery was performed due to the firmness of the inflammatory process. Two Jackson Pratt (JP) drains were placed in the pelvis and right paracolic space. Bacteriological study of the collection showed no germ growth. The patient improved and was discharged after 7 days of admisison; before this a control CT was performed to ensure complete drainage of the periappendiceal collection, as this case presented an atypical scenario for our team.

At 6 months, a follow-up CT (Image 5A, Image 5B) showed an enlarged cecal appendix with a coprolite inside, without acute inflammatory process or collections. Although the patient was asymptomatic, a scheduled laparoscopic appendectomy was suggested due to the persistent enlargement of the appendix, coprolite, and the young age of the patient with a risk of future inflammatory episodes. Laparoscopic appendectomy was performed, revealing a cecal appendix with a significant inflammatory component. The base of the appendix had a diameter of approximately 2.5 cm, requiring the use of a 45 mm blue cutting linear stapler. She was discharged on the third postoperative day without complications.

**Image 5A:**
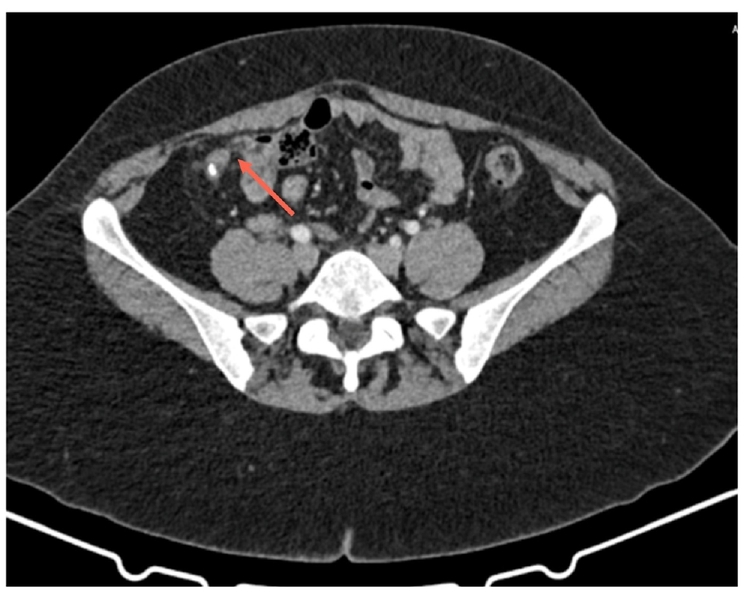
Inflammatory changes with a coprolite in appendiceal lumen.

**Image 5B:**
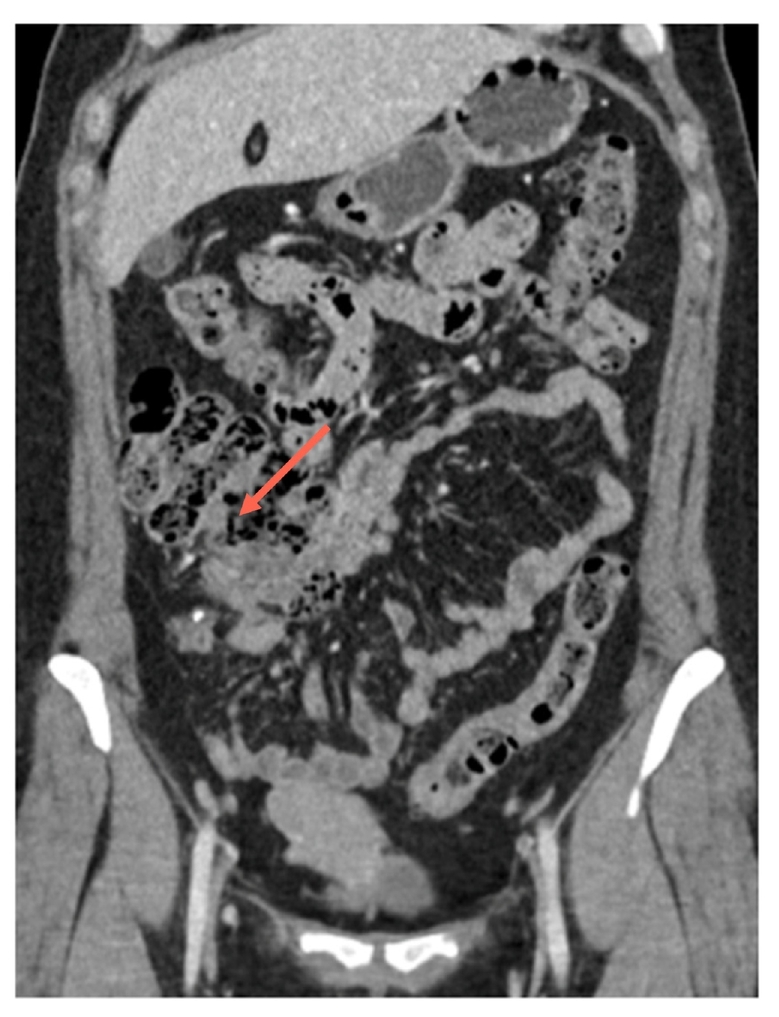
Inflammatory changes with a coprolite in appendiceal lumen.

Histopathological examination showed a cecal appendix with a coprolite inside its lumen and chronic inflammatory process, with endometrial glands lined by cylindrical cells without atypia in the muscular and serosal layers, lined by normal columnar epithelial cells, a classic histological feature of endometriosis; these were surrounded by fusocellular stromal cells with a spindle shape that resembles the endometrial stroma found in the uterine lining with foci of recent bleeding, that indicates hemorrhage associated with endometrial activity in ectopic location; no malignant lesions were found (Image 6A, Image 6B, Image 7).

**Image 6A:**
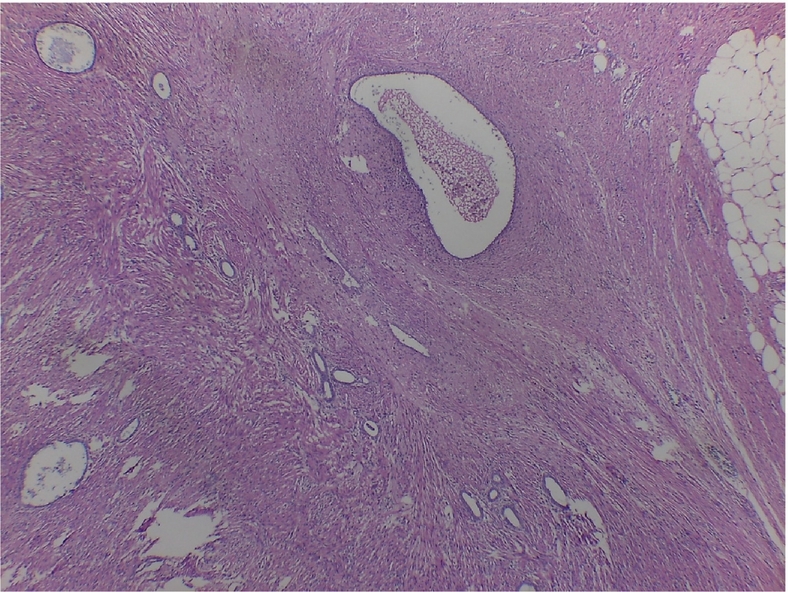
Appendiceal endometrial foci. 10 X.

**Image 6B:**
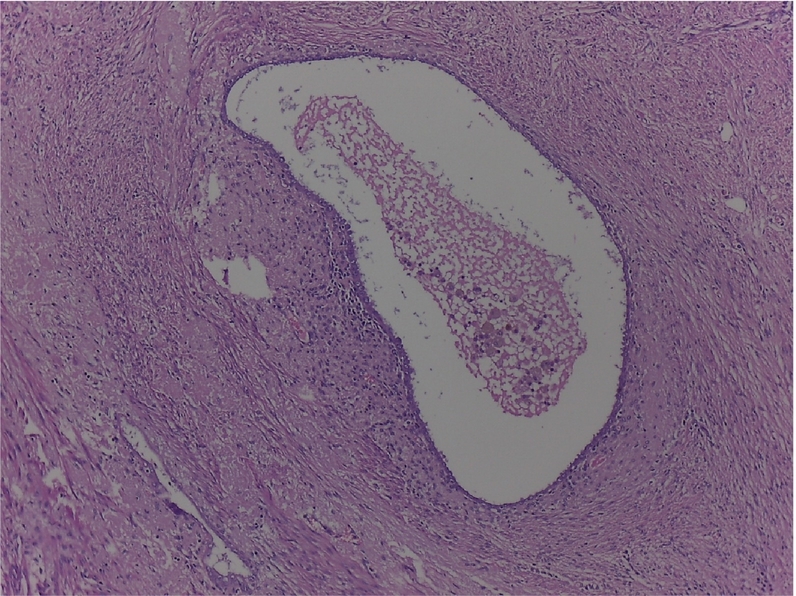
Appendiceal endometrial foci.

**Image 7:**
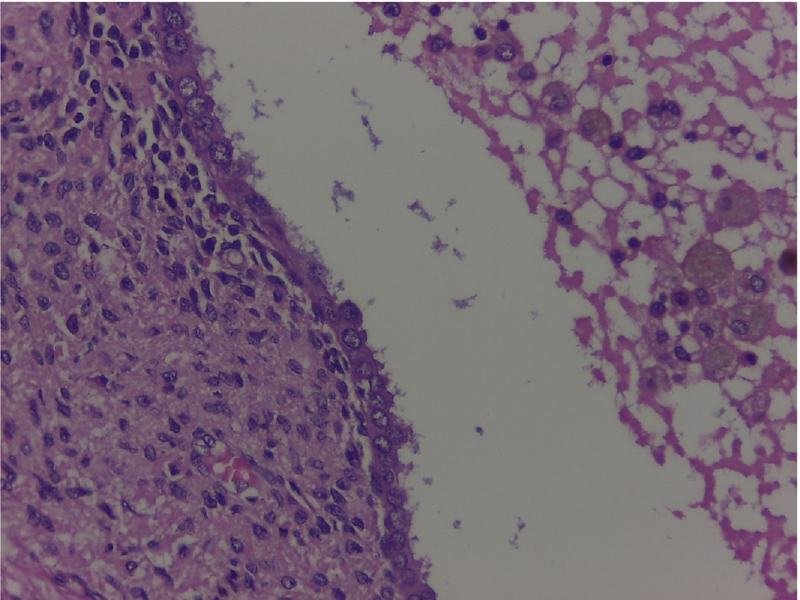
Endometrial foci. Cylindrical epithelium and endometrial stoma. HE 40×.

The patient is currently asymptomatic and under periodic gynecological follow-up.

## 4 Discussion

Endometriosis is defined as the presence of endometrial glands and stroma outside the uterine cavity. It is generally located in the ovaries, fallopian tubes, uterosacral ligaments, anterior and posterior cul-de-sacs, and the uterosacral ligament. Atypical locations include extrapelvic sites such as the gastrointestinal tract, urinary system, soft tissues like the abdominal wall, and thorax, among others [[1], [2], [3]].

Appendiceal endometriosis (AE) was described by Von Rokitansky in 1860. Among all locations in the digestive tract, it is the least frequent, with a prevalence of 0.4 % in the general population and up to 1.7 % in women with known endometriosis [2,6,9,12]. Despite being unusual, AE was the most common histological finding among all gynecological tissue proliferations in 72 appendectomy specimens [16]. In patients with deep endometriosis, the risk of AE is six times higher, with appendiceal involvement reported between 2.5 % and 44.3 % [8].

The pathophysiology of AE is unknown. Some theories suggest ectopic transplantation from the oviduct or retrograde menstruation of endometrial tissue through the tube into the pelvis, supported by the presence of epithelial ectopic tissue similar to endometrium generally found in the muscularis propia or subserosal layer of the appendix. However, some cases of appendiceal endometriosis occur in patients without gynecological disease, countering this theory [2,13].

AE is generally asymptomatic or associated with painful menstrual cycles. Laskou et al. classified AE into four groups based on clinical presentation, which can include cyclical abdominal pain in the lower right quadrant, AA or chronic appendicitis, lower gastrointestinal bleeding, intussusception, abdominal mass, intestinal obstruction, or intestinal perforation, especially during pregnancy [2,6,9,10,[12], [13], [14],^17^,^18^].

AA is one of the most common surgical emergencies, with an incidence of 7–50 cases per 100,000 inhabitants per year [19]. AA results from obstruction of the appendiceal lumen, with the most frequent causes being a coprolite, lymphoid hyperplasia, foreign bodies, parasites, and tumors. Endometriosis is a much less frequent etiology, reported in 0.5 to 2.5 % of cases [2]. Recently, Schrempf et al. reported an incidence of 0.7 % in 2484 consecutive appendectomies for suspected AA [20]. AA caused by endometriosis occurs due to obstruction of the appendiceal lumen by endometrial tissue or bleeding within the seromuscular layer of the appendix, causing edema, obstruction, and inflammation [13].

AE is associated with right-sided endometriomas larger than 5 cm, pelvic involvement, bladder endometriosis, and ileocecal involvement [21]. In our series, both cases had associations not found in the consulted literature. The first case involved AA with right adnexal endometriosis requiring salpingectomy. The second patient presented with an appendiceal phlegmon requiring laparoscopic exploration due to poor response to initial medical treatment and subsequent scheduled appendectomy after 6 months.

Preoperative imaging studies play a crucial role in AA diagnosis. In our experience, abdominal CT with contrast in both cases identified simple or advanced appendiceal inflammation. The high sensitivity and specificity of the method make it the choice for AA diagnosis [12,13,17]. Imaging findings that allowed diagnosis in our series included an increased appendiceal diameter of >7 mm, wall thickening, and periappendicular inflammatory changes with suggestive imaging of a phlegmon. AE may also present on CT as an enlarged appendix surrounded by hypodense masses, focal nodules within the appendiceal body, or central enhancement without obstruction [12,14,22]. Most authors agree that there is no characteristic imaging pattern, which complicates the preoperative diagnosis of this condition [10,[12], [13], [14]].

The recommended treatment for AA is laparoscopic appendectomy [8,12,13,15,17,18]. In cases of AE, if the involvement is greater, partial resections of the cecum and ileum and/or right colectomy might be required [5,23]. It is recommended to evaluate the uterus and adnexa during laparoscopy for endometriosis in patients without a history of the condition. In such cases, the exploration may be accompanied by hemorrhagic fluid and nodularity of the appendix [11,17]. Generally, acute symptoms are resolved after appendectomy, but cyclical abdominal pain may recur due to associated pelvic endometriosis [2,12]. Although controversial, several authors recommend appendectomy during gynecological surgery for deep endometriosis to reduce chronic pelvic pain, the possibility of future AA, and the incidental diagnosis of other conditions [2,6,8,15,21,24].

The definitive diagnosis is histological based on the appendectomy specimen [2,10,13,17]. Organ involvement is more common in the body and tip of the appendix. Generally, the muscular or seromuscular layer is affected, as in our cases, less frequently only the serosal layer is involved, and usually, the mucosa is not compromised [6,7,9,12,13,17,23]. In our experience, both of these cases showed endometrial stroma within the serosa and muscular layer with signs of recent bleeding upon the appendix.

## 5 Conclusion

Acute appendicitis caused by appendiceal endometriosis is an extremely rare condition, with preoperative diagnosis being very challenging despite imaging methods, and the standard treatment is laparoscopic appendectomy. The definitive etiological diagnosis is histopathological. The need for appendectomy during gynecological surgery for deep endometriosis should be seriously considered.

## Methods

This case report has been reported in line with the SCARE criteria [25].

## Ethical approval

Given that this publication is a case report that does not contain identifiable patient information, this publication is exempt from ethical approval by the Institutional Ethics Committee of the Clinica Universitaria Reina Fabiola in Cordoba, Argentina.

There is no source of funding to be stated.

Given that this publication is a case report that does not contain identifiable patient information, this publication is exempt from ethical approval by the Institutional Ethics Committee.

## Guarantor

Abdala Bolcatto, Melisa Erina

Viscido, German Rodrigo

Mandojana, Facundo Ignacio

## Funding

No source to be stated.

## Author contribution

Abdala Bolcatto, Melisa Erina, MD: data collection, writing the paper (First author).

Mandojana, Facundo Ignacio, MD: writing the paper (Corresponding author).

Bruera, Nicolás, MD: data collection.

Doniquian, Alejandro Marcelo, MD: data collection.

Viscido, German Rodrigo, MD: data interpretation, writing the paper.

## Declaration of competing interest

The authors declare no conflicts of interest.

Informed consent for publication was signed and granted by the patients.
